# Supplementary material for: Common variation in meiosis genes shapes human recombination and aneuploidy
Source: Nature. 2026 Jan 21;651(8104):146–53. doi: 10.1038/s41586-025-09964-2 (PMC12960220; doi:10.1038/s41586-025-09964-2)
Supplement: Supplementary file 1 — This file contains Supplementary Methods, Supplementary Note 1, Supplementary Figs. 1–13 and References. [file 41586_2025_9964_MOESM1_ESM.pdf]

nature portfolio

https://doi.org/10.1038/s41586-025-09964-2

Supplementary information

Common variation in meiosis genes shapes human recombination and aneuploidy

In the format provided by the authors and unedited

Supplementary Information

Common variation in meiosis genes shapes human recombination and aneuploidy

Sara A. Carioscia[1]*, Arjun Biddanda[1]*, Margaret R. Starostik[1], Xiaona Tang[1], Eva R. Hoffmann[2], Zachary P. Demko[3], Rajiv C. McCoy[1]**

Supplementary Methods **2**
Data description.. 2
Genotyping, imputation, and quality control 2
Aneuploidy and crossover detection.. 3
Testing associations with maternal age.. 9
Genome-wide and transcriptome-wide association with recombination phenotypes 9
Genome-wide and transcriptome-wide association with maternal meiotic aneuploidy.. 11
Quantifying nucleus-wide covariation in crossovers between euploid and aneuploid embryos 13
Mixed-effect models to contrast crossovers between euploid and aneuploid embryos.. 13
Heritability estimation for recombination and aneuploidy phenotypes.. 14
Electrophoretic mobility shift assay 14
Evolutionary analyses of aneuploidy-associated variants 15
Supplementary Note 1: Modeling negative selection on aneuploidy risk variants 17
Supplementary Figures 20
References.. 29

Supplementary Methods

Data description

Data collection and sampling

IVF samples were collected as part of standard-of-care PGT-A, and data was generated using Natera's CAP-CLIA accredited clinical testing workflow. After fertilization, trophectoderm cells were biopsied from embryos at the blastocyst stage according to the standard protocols of each IVF clinic. Samples were then shipped overnight to the Natera laboratory for PGT-A. Fractions were thawed at 22°C and Arcturus PicoPure Lysis Buffer (Molecular Devices, Sunnyvale, CA, USA) was added to each of the biopsies. The tubes were incubated at 56°C for 1 h and then heat-inactivated at 95°C for 10 min. DNA from the lysed biopsies was amplified using a commercial kit (GE Healthcare, Waukesha, WI, USA) for multiple displacement amplification (MDA). MDA reactions were incubated at 30°C for 2.5 h and then heat-inactivated at 65°C for 5 min. The amplified samples were genotyped using Illumina (San Diego, CA, USA) Infinium II genotyping microarrays (CytoSNP-12 chips) using a modified 24-h protocol, as described previously [58]. Parent buccal samples were collected using MasterAmp Buccal Swabs (Madison, WI, USA). Genomic DNA was isolated from these swabs using Epicentre DNA Extraction Solution (Madison, WI, USA). For parental samples, the standard Infinium II protocol (www.illumina.com) was used. Patient level genetic data and summary metadata (including parental ages, egg and sperm donor statuses, and year of sample collection) were de-identified and provided by Natera to Johns Hopkins for analysis.

Sample overview

After initial quality control (presence of files in dataset, removing families with listed ages of biological parents outside of 18-90 years old), the dataset included 22,850 unique biological mothers with data collected from 2014-2020 (between 2,271 and 4,719 unique mothers per year). Excluding patients that used egg or sperm donors, maternal ages ranged from 20.1 to 55.8 years at the time of collection, with a mean of 36.2 years (**Fig. S1**). Each IVF cycle had a mean of 4.63 embryos (standard deviation = 3.40; range = 1-35). Most pairs of biological parents (17,420) had one recorded cycle, 4,021 had two cycles, and 1,409 had three or more.

Genotyping, imputation, and quality control

We restricted our analysis to samples with genotype data recorded for all array probes. Starting with raw array probe intensity values (x,y), we applied the recommended Illumina normalization procedure:

1. Exclude outliers by being outside the 99th and 1st quantiles of the distribution of x, y, or x/(x+y) across all probes on a chromosome
2. Correct for x and y offsetting from (0,0)
3. Correct for rotational angle from the x-axis (theta)

4. Correct for rotational angle from the y-axis (shear)
5. Scale x,y points by axis-specific mean estimates

The normalization procedure ensures that all intensities are on the same approximate scale prior to genotyping. Following this procedure, we also filtered out ultra-rare variants with global allele frequencies less than 0.1% (15,534 variants removed).

We then used the program optiCall [59] to call genotypes from the normalized array intensities. To avoid population structure driving deviation from Hardy-Weinberg equilibrium, we provided super-population labels derived from k-means clustering of PCA on the raw intensity values (K=3) when calling genotypes. We split each chromosome into segments with approximately 500 genotyped SNPs per chunk. We restricted output to genotypes with posterior probabilities of at least 0.9 (-minp 0.9) and used Hardy-Weinberg equilibrium p-values greater than or equal to the default threshold of 1×10^{-15}. After calling genotypes, we lifted over variants from human genome build GRCh37 to GRCh38, which resulted in the removal of 1,831 variants. Following application of these filters, we retained 275,425 variants across the genome.

In preparation for genotype imputation, we pre-phased the parental genotypes using Eagle v2.4.1 [60] and the combined Human Genome Diversity Project and 1000 Genomes Project (HGDP + TGP) reference panel haplotypes [61]. We then applied genotype imputation with BEAGLE v5.4 [62,63] using the same HGDP + TGP reference panel with default parameters. Each autosome was split into 20 non-overlapping intervals for speed. For the X chromosome, we split the samples into 10 equal groups for more efficient memory management when running imputation. After imputation, we retained variants with dosage r-squared value greater than 0.8 [64].

To evaluate the broad ancestry composition of the parental samples, we combined these genotype data with published data from 2,504 unrelated individuals from the 1000 Genomes Project, restricting to an overlapping set of 257,580 autosomal biallelic variants. We then performed principal component analysis on the combined genotype matrix (**Fig. S2A**). For **Fig. S2B**, samples were labeled based on genetic similarity, using the majority of ancestry labels of the 5 nearest neighbors based on Euclidean distance across the top 20 principal components, scaled by the percentage of variance explained, to reference individuals from the 1000 Genomes Project.

Aneuploidy and crossover detection

A haplotype-copying HMM for aneuploidy detection from allelic intensity data

To model the relationship between array intensities, parental haplotypes, and the underlying ploidy of an embryo chromosome, we formulated a hidden Markov Model (HMM). The hidden states Z_i are tuples representing the maternal $M = (m^{(0)}, m^{(1)})$ and paternal $P = (p^{(0)}, p^{(1)})$ haplotypes that are copied at the ith locus. We detail all the possible hidden states per karyotype class below:

- Nullisomy: $\varnothing$
- Maternal Monosomy: $\{p^{(0)}, p^{(1)}\}$
- Paternal Monosomy: $\{m^{(0)}, m^{(1)}\}$
- Disomy: $\{(m^{(0)}, p^{(0)}), (m^{(0)}, p^{(1)}), (m^{(1)}, p^{(0)}), (m^{(1)}, p^{(1)})\}$
- Maternal Trisomy: $\{ (m^{(0)}, m^{(0)}, p^{(0)}), (m^{(0)}, m^{(1)}, p^{(0)}), (m^{(1)}, m^{(1)}, p^{(0)}), (m^{(0)}, m^{(0)}, p^{(1)}), (m^{(0)}, m^{(1)}, p^{(1)}), (m^{(1)}, m^{(1)}, p^{(1)}) \}$
- Paternal Trisomy: $\{ (m^{(0)}, p^{(0)}, p^{(0)}), (m^{(0)}, p^{(1)}, p^{(0)}), (m^{(0)}, p^{(1)}, p^{(1)}), (m^{(1)}, p^{(0)}, p^{(0)}), (m^{(1)}, p^{(0)}, p^{(1)}), (m^{(1)}, p^{(1)}, p^{(1)}) \}$

For example, for a maternal monosomy at locus i, the model can only copy from either *paternal* haplotype ${p_i}^{(0)}$ or ${p_i}^{(1)}$, since the maternal chromosome is absent. The variable M_i is the allelic dosage (i.e., the number of alternative alleles) of maternal-origin haplotypes copied at locus i; this is analogously defined for the paternal dosage P_i . The variable K_i is the total ploidy (i.e., the size of the tuple defining Z_i). Using these auxiliary variables, we define the emission distribution for the observed allelic intensity at the ith locus, b_i:

$$b_i | M_i, P_i, K_i, \pi_0, \sigma \sim \begin{cases} \mathcal{N}_{[0,1]}\left(\frac{1}{2}, \sigma\right) & , \mu_i = \emptyset \\ \pi_0 \delta_0 + (1 - \pi_0)\mathcal{N}_{[0,1]}(0, \sigma) & , \mu_i = 0 \\ \pi_0 \delta_1 + (1 - \pi_0)\mathcal{N}_{[0,1]}(1, \sigma) & , \mu_i = 1 \\ \mathcal{N}_{[0,1]}(\mu_i, \sigma) & , \text{else} \end{cases}$$

where $\mu_i = \frac{M_i + P_i}{K_i}$ is the expected dosage *conditional* on the parental haplotypes being copied (the case of $\mu_i = \emptyset$ for a nullisomy). Since the range of allelic intensity is between 0 and 1, we model the emission using a truncated normal distribution ($N_{[0,1]}$) or mixture of a point-mass and a truncated normal distribution. The technical noise parameters π_0 and σ reflect 1) the fraction of fully homozygous genotypes lying at the allelic intensity boundaries of 0 or 1 and 2) the standard deviation in the intermediate allelic ratios, respectively.

To complete the HMM definition, we define the transition matrix A. There are two different classes of transitions between hidden states: 1) transitions *within* the same ploidy class and 2) transitions *between* ploidy classes[65]. In the first case, this is likely due to a recombination event, which occurs with rate r. In the second case, inter-ploidy class transitions occur with probability a. We assume throughout that $r >> a$ and set $r = 10^{-8}$ per base pair per generation as an estimate of the genome-wide recombination rate (given that 1 centimorgan ≈ 1 Mbp in humans) and $a = 10^{-10}$ [14]. This means that an inter-ploidy transition is ~100 times less likely than recombination between loci and requires strong evidence over a longer stretch of the chromosome. Using these two variables, A is defined as:

$$\mathbf{A}_{j,k} = \begin{cases} r & , K_i = K_j \\ r \times a & , K_i \neq K_j \end{cases}$$

where K_j is the ploidy count of latent state j and $A_{i,i} = 1 - \sum_{j \neq i} A_{i,j}$. The full transition probability is therefore:

$$P(z_{i+1} = k \mid z_i = j) \propto A_{j,k} d_{i,i+1},$$

where $d_{i,i+1}$ is the physical distance in base pairs between locus i and $i+1$. Using the forward algorithm to compute the likelihood of the data $\bar{b}$, we obtain maximum-likelihood estimates $\widehat{\pi_0}$ and $\hat{\sigma}$ using the bounded BFGS algorithm for numerical optimization [66]. Using the MLE parameters $\widehat{\pi_0}$ and $\hat{\sigma}$, we calculate the posterior probability of being in each state γ_{z_i} at locus i via the forward-backward algorithm [66]. We calculated the posterior probability of a given ploidy context as the scaled posterior probability of ploidy across all sites on the chromosome. For the case of a disomy:

$$P(disomy \mid M, P, b) = \frac{1}{L} \sum_{i}^{L} \sum_{z_i \in disomy} \gamma_{z_i}$$

The posterior probability of the other ploidy classes is similarly calculated. Following the calculation of the posterior probability of each ploidy configuration, we assign the ploidy configuration with the maximum posterior probability as the karyotype for the chromosome in that embryo. Downstream, we use the maximum posterior probability > 0.9 as a threshold for high-confidence whole-chromosome aneuploidy.

Sex chromosome aneuploidy detection

To accommodate calling sex chromosome aneuploidies, we modify the hidden states of the HMM for the X chromosome:

- Loss of X: $\emptyset$
- Single paternal copy of X: $\{p^{(0)}\}$
- Single maternal copy of X: $\{m^{(0)}, m^{(1)}\}$
- Disomic Bi-parental X chromosome inheritance: $\{(m^{(0)}, p^{(0)}), (m^{(1)}, p^{(0)})\}$
- Uniparental Disomy of maternal X chromosome: $\{(m^{(0)}, m^{(0)}), (m^{(0)}, m^{(1)}), (m^{(1)}, m^{(1)})\}$
- Trisomy of X chromosome: $\{(m^{(0)}, m^{(0)}, p^{(0)}), (m^{(0)}, m^{(1)}, p^{(0)}), (m^{(1)}, m^{(1)}, p^{(0)})\}$

To adjust the model for the Y chromosome we only use two hidden states:

- Loss of Y: $\emptyset$
- Presence of Y: $\{p^{(0)}\}$

We used the same transition model as was used for the autosomes: a recombination rate of 10^{-8} per base pair per generation and inter-ploidy transition rate of 10^{-10}. Posterior probabilities of chromosome-wide ploidy states are obtained by collapsing the results of the forward-backward algorithm. Karyotype status is assigned as the maximum posterior probability, and we similarly filter on posterior probability > 0.9 to obtain high-confidence whole-chromosome aneuploidy calls.

Performance evaluation of aneuploidy detection

To evaluate the performance of our method for estimating karyotypes, we simulated array intensity data assuming various whole-chromosome ploidy states. Specifically, we simulated parental haplotypes with 4,000 SNPs along a contig of 35 Mbp (a close approximation to the real data on chromosome 22 for parental haplotypes). To reflect ascertainment of alleles in array data, we drew parental alleles under Hardy-Weinberg equilibrium from the distribution of global allele frequencies of variants on the Illumina HumanCytoSNP array from the 1000 Genomes Project [67]. To account for switch error rates consistent with population-based phasing, we simulated a switch-error rate of 3% across parental haplotypes, representing an upper-bound of expected phasing errors [68]. Under the parameters π_0, σ , we simulated 40 replicates each of nullisomy, monosomy, disomy, and trisomy. As the copying probability is symmetric across the sexes, we only focus on simulating monosomy and trisomy of paternal origin for performance evaluation.

Under the case of true meiotic aneuploidies, across the entire range of parameters the precision and recall of the current method are > 0.95 across all categories simulated, indicating high accuracy for detection of aneuploidies while accounting for genotyping array-specific noise (**Fig. S3**).

Early embryos may also be affected by mosaic aneuploidies, where only a fraction of the cells in the biopsy are aneuploid and others are disomic [69,70]. To assess the potential confounding impacts of mosaic aneuploidies, we simulated both 5 and 10-cell biopsies, where the expected proportion of cells containing a specific aneuploidy is p_{mosaic} . We simulated 20 replicates across p_{mosaic} from 0% to 100% of the cells containing an aneuploid set of chromosomes. Each mosaic cell that is simulated represents a single focal aneuploidy (either monosomy or trisomy). We then averaged the allelic intensities across all the cells (disomic and non-disomic) to create the vector of allelic intensities for inference.

We find that simulated mosaic aneuploidies tend to exhibit different effects depending on whether the mosaic aneuploidy is a monosomy or trisomy and their fraction in the dataset. For mosaic trisomies, once the cell fraction increases to approximately 50%, we observed confident trisomy calls along the whole chromosome irrespective of genotyping array noise (**Fig. S10**). Monosomies behave slightly differently, where mosaic monosomies with a cell fraction between 10% and 80% tend to be confidently called as trisomies (**Fig. S10**). This is because a mixture of monosomic and disomic chromosomes create modes in the distribution of allelic intensities that are similar to those of a true meiotic trisomy (e.g., peaks in allelic intensity ratios at ⅓ or ⅔). When the cell fraction of monosomy is > 80%, we find that a monosomy is confidently called with a posterior probability > 0.9. Therefore, while our method performs well for detection of meiotic aneuploidies, certain forms of mosaic aneuploidy may impact performance, motivating additional filters.

Filtering mosaic aneuploidies

To statistically separate mosaic (mitotic-origin) from meiotic aneuploidies, we exploit two well-characterized signatures of mitotic aneuploidies. The first signature is that mitotic trisomies only display *single parental homologs* (SPH) from a given parent, whereas meiotic-origin trisomies contain *both parental homologs* (BPH) from one of the parents and thus three genetically distinct parental haplotypes [6,13,71]. The second signature is that mitotic aneuploidies exhibit no strong relationship with maternal age [19]. For trisomic calls made under the HMM model, we estimate the posterior probability of a chromosome being in a BPH vs. SPH state along the entire length of the chromosome. It should be noted that the BPH vs. SPH is not a perfect indicator of meiotic vs. mitotic trisomies (or mosaic monosomies), as tracts of BPH may exist in distal regions of chromosomes, outside of the range of the array [71]. Similarly, meiosis II errors with no recombination may manifest as SPH trisomies. In order to distinguish mitotic from meiotic aneuploidies, we investigated at what level of BPH the maternal age effect becomes significant.

For each confidently called trisomy (posterior probability > 0.9), we calculated the posterior probability of BPH and considered every trisomy call with $P(BPH \mid Data, \Theta) < \alpha$ a mitotic-origin aneuploidy. We then ran a binomial regression for the number of inferred mitotic aneuploid embryos against maternal age and tested at what level α the effect-size for the linear effect becomes significantly positive (**Fig. S11**). We tested across a grid of 100 points for α from 0 to 1 to determine when $\widehat{\beta_{age}} - \widehat{2s_{age}} > 0$, which are the estimated effect size and standard error for the age effect, respectively. We expect at higher thresholds of BPH that meiotic aneuploidies will begin to be included in this filter, making the age effect non-zero. We find that a posterior filter of $P(BPH \mid Data, \Theta) < 0.340$ maintains the null effect of maternal age for inferred mitotic aneuploidies (**Fig. S11**). This filter likely captures mitotic-origin trisomies affecting all cells of a given biopsy, as well as mosaic trisomies and monosomies of medium cell fractions.

Segmental aneuploidy detection and filtering

To prioritize aneuploidies affecting whole chromosomes, we developed a pipeline to identify sub-chromosomal (i.e., "segmental") aneuploidies using the HMM output and exclude these chromosomes from downstream analyses. Using the maximum *a posteriori* (MAP) path through the HMM, we identify changepoints in the path that support different karyotypic states (i.e., inter-ploidy transitions). Smaller segmental aneuploidies may potentially be present on longer chromosomes but may not represent enough of the MAP path across the chromosome to shift the total posterior across all sites to be < 0.9, which we use to assign whole-chromosome aneuploidy status.

We identify changes in the MAP path from the majority ploidy of the chromosome and determine whether it is a segmental aneuploidy based on 1) whether it contains at least 100 SNPs, 2) whether the local posterior within the segment supporting its ploidy assignment is > 0.8 across the segment, and 3) whether the segment is at least 5 Mbp long. We find that this set of filtering criteria has reasonable power for identifying simulated segmental aneuploidies that are > 5 Mbp in length with a precision > 90% across different scales of embryo-level noise (**Fig. S12**). We

exclude all chromosomes with segmental aneuploidies called in this way from our current analyses of whole-chromosome meiotic aneuploidy. Conceptually, many changepoints occurring in the MAP path indicate either 1) biopsy errors or 2) potential mosaic aneuploidies. In either case, we expect that both of these occurrences will prevent an aneuploidy from reaching a chromosome-wide posterior probability > 0.9.

Calling crossover recombination events in PGT-A data

To identify crossover events, we used a previously defined heuristic approach, which uses switches in the assignments of informative variants from each parent to designate the endpoints of a crossover in a template embryo relative to its sibling embryos [22,23,72,73]. For each chromosome, we restrict our analysis to only families that have ≥ 3 disomic embryos for that chromosome (noting that embryos may have aneuploidies occurring on other chromosomes).

Previous methods relied on *called genotypes* for siblings, which are not reliably available for PGT-A data. Therefore, we adapted the method to consider the likelihood that the same parental allele is transmitted to the non-template sibling embryo. Our approach is based on the likelihood for the embryo genotype array intensity, conditional on the parental genotypes (P_i, M_i for paternal and maternal, respectively) at site i: $L(b_i \mid M_i, P_i, \pi_0, \sigma)$. In this case we also use the MLE estimates of $\widehat{\pi_0}, \hat{\sigma}$ for the embryo under consideration. To illustrate, consider two informative biallelic SNPs (a, b) for a maternal crossover event—where the maternal genotypes are heterozygous and the paternal genotypes are homozygous—at which we can compute the following likelihood of the *same allele being transmitted* to both the template (t) and a single non-template embryo (nt):

$$P(Same\ Allele) = \prod_{i \in (a,b)} \sum_{x \in (0,1)} L({b_i}^t \mid M_i = x, P_i, {\pi_0}^t, \sigma^t) \times L({b_i}^{nt} \mid M_i = x, P_i, {\pi_0}^{nt}, \sigma^{nt})$$

The likelihood for *different alleles being transmitted* from the maternal side at (a, b) for the template and non-template embryos is:

$$P(Different\ Allele) = \prod_{i \in (a,b)} \sum_{x \in (0,1)} L({b_i}^t \mid M_i = x, P_i, {\pi_0}^t, \sigma^t) \times L({b_i}^{nt} \mid M_i = \neg x, P_j, {\pi_0}^{nt}, \sigma^{nt})$$

Thus, for every pair of t, nt embryos within the family, we can determine whether a crossover event occurred in the transmission from the mother to the template embryo between a and b by evaluating the log-likelihood ratio $llr_{\{a,b\}} = log(P(Same\ Allele)) - log(P(Different\ Allele))$. Following previous work, we create a binary set of “switch indicators" of $llr_{\{a,b\}} < 0$ or $llr_{\{a,b\}} > 0$ between each pair of informative markers to isolate switch points indicative of crossover events [23].

To leverage multiple sibling embryos and account for noise on the genotyping array, we apply two further refinement steps to isolate crossovers. The first step considers up to five adjacent pairs of informative sites and determines whether the number of switches within the switch

cluster is odd, since an even number of switches (very close and nearby crossover) can be indicative of genotyping error [23]. The second filter restricts crossovers to be supported by the majority of the sibling embryos to ensure that multiple meioses from the parental individual support a crossover call.

Testing associations with maternal age

To test the relationship between maternal meiotic aneuploidy and maternal age (**Fig. 1D**), we summed counts of aneuploid and euploid embryos over cycles from a given set of biological parents and fit a quasibinomial generalized linear models. We deemed this model family most appropriate given the count-based nature of the binary outcome data (embryos affected versus unaffected by maternal meiotic aneuploidy) with dispersion exceeding binomial expectations. We excluded cases using egg donors from this analysis, as the ages of the donors were unavailable. We then fit a series of nested models with increasingly complex polynomial predictor terms for maternal age, selecting the best model by 10-fold cross-validation. Specifically, we evaluated the mean binomial deviance across held-out folds for increasingly models ranging from polynomial degrees of 0 (i.e., no maternal age effect) to 6 (i.e., a sixth-degree polynomial term). We observed that beyond the quadratic term, the change in binomial deviance was negligible, and we opted to retain the quadratic model according the "one standard error rule", which selects the most parsimonious model within one standard error of the minimum cross-validation error [74] (**Fig. S4**).

Genome-wide and transcriptome-wide association with recombination phenotypes

Recombination phenotypes were defined in the following categories on a sex-specific and joint basis:

1. Crossover count
   For each meiosis from a given parent, we estimated the total number of crossovers across all autosomes. We define the phenotype as the mean number of autosomal crossovers across all euploid embryos.

2. Hotspot Occupancy
   We first defined hotspots as regions where the sex-averaged genetic map has a relative recombination rate > 10, following previous definitions [14]. We then estimate the *fraction* of crossover intervals that intersect with hotspots after permuting crossover intervals [23]. The phenotype is an approximation of the maximum likelihood estimate of the fraction of crossovers of a specific parental origin (across all meioses observable for that parent) that occur in hotspots.

3. Replication Timing
   Replication timing was derived from measurements in 300 induced pluripotent stem cell (iPSC) lines [75]. Coordinates were lifted over to GRCh38. Using the average replication timing across all 300 iPSCs, we used linear interpolation to estimate the replication

timing at each crossover interval midpoint. Replication timing for an individual was estimated as the mean replication timing value across all corresponding crossover intervals.

4. GC Content
   Guanine-cytosine (GC) content at each estimated crossover location was calculated using the GC percentage track in five base pair increments from the UCSC browser in GRCh38 coordinates. We estimated the GC content at each crossover location using the average GC content within 500 bp upstream and downstream of the midpoint of the crossover interval. GC content for each individual was estimated as the average GC content across all crossovers attributed to that individual, across all corresponding meioses.

All phenotypes were inverse-rank normal transformed within each sex-specific study and jointly and tested using REGENIE [76]. All association testing included age, number of sibling embryos assayed, the average estimated π_0 and σ parameters corresponding to the embryos from a given parental pair, and 20 genetic principal components across all the parental individuals as covariates. For joint testing of maternal and paternal phenotypes, we also included sex as a covariate.

We conducted linkage disequilibrium (LD) clumping (plink2 --clump-r2 0.1 --clump-kb 1000) of association results to identify a set of approximately independent variants per phenotype. To map the lead variant within each locus to a gene, we assigned the closest gene and reported the distance to the gene boundary in GENCODE v37 (**Table S2**). To evaluate potential novelty of association results, we evaluated replication at two scales: 1) whether a specific lead variant in a locus replicates, and 2) whether a specific gene is also found to replicate in a previous GWAS of recombination phenotypes [14].

We performed GWAS across these 4 recombination phenotypes, stratifying by parental origin as well as considering both parents together (12 total GWAS). We identified 42 approximately independent loci ($r^2 \leq 0.1$) exceeding the threshold of genome-wide significance ($p < 5 \times 10^{-8}$), implicating 15 unique genes, all of which replicate previous findings in the literature [14] (**Table S3**).

TWAS for each recombination phenotype was conducted similarly to the TWAS for aneuploidy (see Methods). We applied a linear model using the glm function (family=gaussian) within R (version 4.3.3[77]) to test the association between the predicted expression levels of genes and each recombination phenotype on a sex-specific and joint basis. Covariates in the model were the same as those used in the GWAS. A combined TWAS p-value was computed for each protein-coding gene across all tissues using the ACAT [78,79] method followed by Bonferroni correction for multiple testing (p = 0.05 / 16,685 unique protein-coding genes = 3×10^{-6}).

Genome-wide and transcriptome-wide association with maternal meiotic aneuploidy

Filtering copy number calls

The aneuploidy phenotype is based on karyoHMM (see Methods), which outputs a posterior probability for each copy number state for each chromosome. We only consider chromosomes that have a posterior probability > 0.9. Any chromosome that does not reach that threshold is excluded from the analysis, though remaining chromosomes from the same embryo are still considered. Embryos with a nullisomy call for 5 or more chromosomes were deemed to have inadequate or missing data and are excluded from analysis. We additionally required a minimum Bayes factor of 2, indicating that a given call has at least twice the support of the next most likely copy number state.

The karyoHMM method also estimates the standard deviation of the B-allele frequency (BAF) distribution conditional on parental genotypes, $\hat{\sigma}$, which quantifies the experimental noise in PGT biopsy data (see Methods). To reduce potential experimental noise, we excluded embryos with an average $\hat{\sigma}$ outside 3 standard deviations from the cohort-wide mean. The karyoHMM method also identifies segmental aneuploidies (copy number gains/losses only affecting a portion of a chromosome). To ensure our phenotype was focused on whole-chromosome gains and losses, we removed any chromosome that was affected by a segmental aneuploidy, defined as a stretch greater than 5 Mbp with more than 100 SNPs and a greater than 80% posterior probability for a copy number other than disomy (see Methods).

Genome-wide association study

To select a set of unrelated individuals for association testing, we used KING [80] to exclude all first or second degree relatives. 5,430 female individuals were present in the dataset multiple times, indicating multiple IVF cycles. To define discovery and test sets, we calculated a weighted mean age and total embryo count by merging families that underwent multiple cycles of IVF. After removing duplicate individuals, we randomly assigned 85% of the mothers to the discovery set, verifying that the distributions of maternal age and number of day-5 embryos were similar between the discovery and test sets. This procedure resulted in the assignment of 19,529 unique mothers to the discovery set and 3,447 unique mothers to the test set. We then propagated these assignments to the corresponding partners.

The aneuploidy phenotype was defined on a per-cycle basis. Cycles were inferred based on metadata provided by Natera, in which each unique set of biological parental ages was interpreted to define one IVF cycle. Most often, a single casefile identifying number (casefileID) comprised a single IVF visit. In situations where ages were provided for the mother but not the father or embryos, the ages listed for a mother were propagated to other samples within the same casefileID. For cases that used an egg or sperm donor, the provided parental ages were those of the individuals undergoing IVF rather than the individual providing the egg or sperm. Therefore, egg donors were assigned the average age of egg donors, 25 years, based on a published retrospective study of egg donors spanning years overlapping our study [81]. Sperm

donors were similarly assigned the average age of sperm donors, 27 years, based on a published study [82].

An embryo was categorized as aneuploid if it possessed between 1 and 5 autosomes assigned as maternal-origin aneuploid by karyoHMM. To conduct the association test for aneuploidy, we applied a generalized linear mixed-effect model (implemented via glmer function from the R package lme4, version 1.1.35.5 [78]), where patient ID was included as a random effect grouping factor to account for the fact that 23.76% of female individuals had multiple IVF cycles. We used a binomial family, as the phenotype is encoded as the counts of aneuploid and euploid embryos per cycle. We used the first 20 genotype principal components (PCs), maternal age, paternal age, egg donor status, and sperm donor status as fixed effect covariates. To obtain the PCs, we applied a principal components analysis (PCA) using PLINK (version 1.9) to the parental genotypes (output from optiCall) for all autosomes.

To test whether the incidence of female meiotic aneuploidy is individual-specific, we fit a quasi-binomial generalized linear regression model to the counts of embryos affected versus unaffected with maternal meiotic-origin aneuploidy (combining across cycles per set of biological parents), including a quadratic term to model the relationship with maternal age. Three female individuals with maternal age greater than 50 years were excluded from this analysis, as they were observed as high-leverage outliers based on Cook's distance (mean D = 0.22). We then simulated new counts of affected and unaffected embryos from the fitted model for the same sample (n = 1,000 simulations), but assuming no overdispersion (i.e., dispersion parameter (φ) = 0). Dispersions were calculated as the sum of squared Pearson residuals, divided by the residual degrees of freedom.

We used the LDproxy tool from LDlink[83] to evaluate LD between GWAS lead SNPs and other potential causal variants within the genomic region. Specifically, we computed pairwise LD between a given query variant and other variants in a ±500 kbp window using the genotype data from high-coverage sequencing of European population samples from the 1000 Genomes Project[84], aligned to reference genome build GRCh38. The history of the variant at the *SMC1B* locus was estimated by comparison with great ape [85] and Neandertal genomic data [86–88].

Transcriptome-wide association studies (TWAS) of maternal meiotic aneuploidy

We performed TWAS by using the imputed parental genotype data (see Methods) to predict genetically regulated gene expression across each of 49 tissues. The published 49 tissue-specific multivariate adaptive shrinkage in R (MASHR) prediction models were trained on GTEx v8 expression data [89,90].

For each gene, we then applied a linear mixed-effects model as described for GWAS above with the same covariates, but here evaluating the association between counts of aneuploid versus euploid embryos and predicted gene expression. We repeated this procedure for each tissue, then combined the single-tissue TWAS p-values using the Aggregated Cauchy Association Test (ACAT) [79]. Multiple hypothesis testing correction was performed using a

Bonferroni correction for the number of protein-coding genes across all tissues ($p = 0.05$ / 16,685 unique protein-coding genes = 3×10^{-06}).

We investigated the extent to which co-regulation might lead to multiple association signals at a TWAS locus by plotting the expression of pairs of genes across individuals from published GTEx v8 expression data [26].

Quantifying nucleus-wide covariation in crossovers between euploid and aneuploid embryos

To establish evidence of per-nucleus covariation in crossover counts per embryo, we used simulation and variance decomposition routines from previous research on individual gametes [29]. To compare against independent simulations, for each of the 46,861 euploid embryos with estimated crossovers, we created an independent crossover count by drawing each autosomal crossover count from the full pool of embryos assayed for that chromosome, restricted to the appropriate parent. We observe that for both maternal and paternal crossovers, there is substantial overdispersion in crossover counts, consistent with previous findings in oocytes [29] (**Extended Data Fig. 7**).

To decompose the per-embryo variance in crossovers, we turn to a decomposition of the variance in crossover number, where the total variance (*A*) is decomposed into the independent component of variance on a chromosome (*B*) and the covariance *between* chromosomes in crossover count (*C*):

$$Var(\sum_{k=1}^{n} CO_k) = \sum_{k=1}^{n} Var(CO_k) + \sum_{i \neq j}^{n} Cov(CO_i, CO_j)$$

A ratio of *C/A* >> 0, which we term inter-chromosomal covariance (ICC), implies that a substantial fraction of the variance in autosome-wide crossover counts is contributed by positive covariance between crossovers on individual chromosomes (**Table S6**).

To compare with classical statistical estimators, we also computed the intra-class correlation (also ICC) in crossover counts stratified by parental origin, where the classes are defined as the per-chromosome crossover counts and are grouped by individual embryo identifiers. The intra-class correlation in this case is significantly non-zero for both maternal (0.176; $p < 10^{-100}$) and paternal crossovers (0.088; $p < 10^{-100}$).

Mixed-effect models to contrast crossovers between euploid and aneuploid embryos

To compare the crossover counts between euploid and aneuploid embryos on their corresponding disomic chromosomes, we used mixed-effect models that include nested random effects for both the parental individual and the embryo in question. For crossover counts, we use a Poisson mixed-effect model with a log link function:

$$Y_{chr,par,k} \sim \mu_{chr} + \gamma_{par,k} + A_k + C + \epsilon_{par,chr,k} ,$$

where $Y_{chr,par,k}$ is the crossover count on a specific chromosome chr for parent par and embryo index k. The Poisson model was deemed most appropriate given the count-based nature of the phenotype and the close adherence to Poisson distributions (**Fig. 1E**). The random effect $\gamma_{par,k}$ is a nested random effect for embryo-specific variance nested within parent-specific variance components in crossover counts (i.e., (1 | par / k) in R). Fixed effects included the expected rate of crossovers per chromosome (μ_{chr}) using the total centimorgan distance as a fixed covariate, an indicator of whether the embryo contained an inferred maternal meiotic aneuploidy (A_k), and female individual-specific covariates (C) which include ancestry principal components, maternal age, and average estimates of embryo noise parameters from karyoHMM.

The model was fit using REML in the lme4 (version 1.1.35.3) package in R. For reporting results, we use the estimated marginal means (via the emmeans package) for the binary indicator A_k of an inferred maternal meiotic aneuploidy affecting the kth embryo.

To assess the effect of patient-specific crossover rates on aneuploid embryos, we first extracted the estimated mean parental random effects (across both euploid and aneuploid embryos) from the above Poisson mixed-effect model ($\widehat{\gamma_{par}}$). We then used the per-patient crossover rates in a binomial mixed-effect model to test whether donor-specific crossover rates were associated with the proportion of aneuploid embryos, while adjusting for principal components, parental ages, parental age squared, donor status, and the genotype for each of the significant SNPs included in our phenome-wide association study.

Heritability estimation for recombination and aneuploidy phenotypes

The SNP heritability for maternal meiotic aneuploidy and recombination phenotypes was estimated using LD-score regression with LD scores computed from our sample genotype data [91]. SNP-heritability for age at menopause and age at menarche was estimated using LD-score regression using LD scores computed on individuals assigned to the EUR continental group within the HGDP + TGP reference panel [61] as well as summary statistics from the ReproGen consortium [43,44]. SNP heritability estimates for body mass index and height were downloaded from the pan-UK Biobank project [92]. Heritability estimates for infertility traits were accessed from published data [48]. Clinical definitions of infertility followed those from Venkatesh et al. (see their Supplement 3, Table 1 for clinical criteria) [48].

Electrophoretic mobility shift assay

Using MAST from the MEME suite [93], we found that the reference genome sequence surrounding a fine-mapped eQTL of *SMC1B* (rs2272804; **Fig. 3D**) is a predicted binding motif for the transcription factor ATF1, whereas the sequence including the alternative allele is not. To test this potential reduced binding activity *in vitro*, we conducted an electrophoretic mobility shift assay (EMSA) with four DNA sequences: the sequence of *SMC1B* surrounding the putative ATF1 binding site and containing the REF allele of rs2272804, the same sequence but

substituting the ALT allele of rs2272804, an ATF1 consensus binding sequence (positive control [94]), and a sequence with no predicted ATF1 binding activity (negative control).

The forward sequence of the DNA fragments are recorded here, with the ATF1 motif bolded and underscored. The variant base is the last position of this motif (C → A).

- Sequence centered on rs2272804 with REF allele (30 bp)
    - 5′-TGTACCTCTGCGG**CGTCAC**TGGGAGCCCGA-3′
- Sequence centered on rs2272804 with ALT allele (C → A) (30 bp)
    - 5′-TGTACCTCTGCGG**CGTCAA**TGGGAGCCCGA-3′
- Positive control: ATF/CRE consensus (30 bp) from the Epstein-Barr virus *LMP1* gene promoter
    - 5′-TCTAGCTCTCTGA**CGTCAG**GCAATCTCTGA-3′
- Negative control: HSPA1A (hsp70) promoter (35 bp)
    - 5′-ATCGAGCTCGGTGATTGGCTCAGAAGGGAAAAGGC-3′

DNA oligonucleotides were ordered from IDT for both the forward and reverse strand, with the forward oligonucleotides labeled at the 5’ with FAM. Oligonucleotides were dissolved in duplex buffer (100 mM potassium acetate; 30 mM HEPES, pH 7.5) and complementary oligonucleotides were annealed to double-strand fragments in a thermocycler by heating up to 95°C for 5 min, then ramping down to 20°C at 5°C/min.

EMSA was used to determine the K_D of ATF1 to DNA fragments. 5nM DNA were mixed with various concentrations of recombinant ATF1 protein (SinoBiological, A09-54G) in the presence of 50 ng poly(dI-dC) (Thermo Fisher Scientific, 20148E) in a 10 µl reaction containing 25 mM HEPES pH 7.5, 50 mM KCl, 50 mM NaCl, 5 mM MgCl2, 5% glycerol, 1 mM DTT, 0.01% IGEPAL, 0.25mM TCEP, and 250 ng/µl BSA. The reactions were incubated at 37°C for 30 min, then analyzed by electrophoresis on 2% agarose gel run in 0.2x TB buffer (17.8 mM Tris and 17.8 mM boric acid) at 100V for 45 min. Gels were scanned on a Typhoon 5 Variable Mode Imager (GE Biosciences), and bands were quantified using the Image Studio software (LICORbio) to calculate F_{bound} (fraction of DNA bound) as $I_{bound}/(I_{bound} + I_{unbound})$, where I is the intensity of the corresponding band.

The F_{bound} in each reaction was plotted versus the concentration of protein, and the data were fit with the binding equation $F_{bound} = B_{max}([\text{ATF1}]/(K_D + [\text{ATF1}]))$ by non-linear regression to estimate the value of K_D, where $B_{\max}$ (the fraction bound at which the data plateaus) was assumed to be 1 [95].

Evolutionary analyses of aneuploidy-associated variants

To estimate the frequency trajectory of the derived allele of rs227804 as a function of time, we first drew posterior samples from the ancestral recombination graph (ARG) using SINGER [96]. We estimated local genealogies using phased genotypes from individuals from the European

continental group based on uniformly processed whole-genome sequencing data from the 1000 Genomes Project and Human Genome Diversity Project [61].

SINGER estimation was performed using all variants within a 4 Mbp window, a default mutation rate of 1.25×10^{-8} per base pair per generation, and an effective population size of 10^4. We then took 100 posterior samples from the ARG and estimated the allele frequency of a variant at a specific node as the fraction of lineages in the tree that are carriers of the allele, repeating this estimation for each genealogy containing the focal variant of interest. The mean and standard error allele of the frequency across all posterior samples was computed in 30 log-spaced bins of time in generations.

BetaScan2 statistics were downloaded from the original authors' repository [97] and plotted in a 250 kbp window surrounding the lead GWAS hit for maternal meiotic aneuploidy at the *SMC1B* locus (rs6006737). For ease of visualization, we limited plotting to positive standardized beta statistics, as negative values have no clear interpretation in the context of balancing selection.

Supplementary Note 1: Modeling negative selection on aneuploidy risk variants

To model the effect of the aneuploidy risk allele on the potential number of euploid embryos (C) produced over a given maternal reproductive timespan, we formulated a model of maternal meiotic aneuploidy conditional on maternal genotype, G:

$$E[C \mid G] = \int_a^b \big(1 - f(t, G)\big)\, dt \ ,$$

where the function $f(t)$ reflects the probability of maternal meiotic aneuploidy as a function of maternal age in years (t), including the effect of the aneuploidy risk genotype (**Fig. 2B**), defined as:

$$f(t, G) = \ logit\big(c \ + \ \beta_{age} t \ + \beta_{age2} t^2 + \beta G\big) \ ,$$

where the parameters estimated from our study are as follows (**Fig. 1C**):

	Estimate	Standard Error
c	-1.20808	0.01456
β_{age}	1.08066	0.01238
β_{age2}	0.21276	0.01015
β	0.06682	0.01178

While the units for the reproductive window for $E[C|G]$ are in years, the units for t in the function f are standardized as $\hat{t} = \frac{t-\mu}{\sigma}$, where $\mu = 35.952, \ \sigma \ = \ 4.892$ based on the empirical maternal ages in the Natera cohort.

The relative fitness of the genotypic classes is the ratio of their expected number of embryos that lack maternal meiotic aneuploidies that could potentially be produced over the course of the female reproductive timespan $[a, b]$:

$$W_{proxy} = \frac{E[C \mid G = 1]}{E[C \mid G = 0]} = \frac{\int_a^b \big(1 - f(t, G = 1)\big)\, dt}{\int_a^b \big(1 - f(t, G = 0)\big)\, dt}$$

We refer to this value as W_{proxy} to denote that it represents an imperfect proxy for fitness. Due to the binomial model (with logit link) of the quadratic relationship between maternal age and incidence of maternal meiotic aneuploidy, W_{proxy} is affected by the bounds of the reproductive window, particularly the upper bound (b; **Fig. S9**). A geometric interpretation of W_{proxy} can be

obtained by comparing the areas over the curve within a given maternal age range between genotypic classes (**Fig. 2B**).

As our naming conventions imply, it is important to consider that the W_{proxy} is not equivalent to fitness, as numerous other genetic and environmental factors modulate realized fitness. This is consistent with our observation of low $\widehat{h^2_{SNP}}$ from LDSC for maternal meiotic aneuploidy incidence, as well as the fact that maternal age explains substantially more variance in aneuploidy incidence than genetic variation. Both points suggest that depending on the strength of correlation between the expected number of embryos lacking meiotic-origin aneuploidies and fitness, the fitness effect of the allele may be reduced compared to the fitness effect based on the proxy. Assuming a linear model of the following form:

$$W = \beta_0 + \beta_1 W_{proxy} \ ,$$

and further assuming that $\beta_0 = 0$ (i.e., when W_{proxy} is zero, W is also 0), the slope of the linear relationship (β_1) can be interpreted as:

$$\beta_1 = r \, \frac{\sigma_{W_{proxy}}}{\sigma_W} \, ,$$

where r is the correlation coefficient between W and W_{proxy}, and σ is the standard deviation of each fitness definition respectively. We can alternatively define the relationship between the realized fitness W and W_{proxy} as:

$$W = \frac{\alpha E[C \mid G = 1] + (1 - \alpha)E[C \mid G = 0]}{E[C \mid G = 0]} = \alpha W_{proxy} + (1 - \alpha)$$

In the equation above, the variable α represents a "scaling factor" for the realized fitness effect, whereas $\alpha \rightarrow 0$, the fitness of a heterozygous carrier of the aneuploidy risk allele becomes the same as a non-carrier. This scaling factor is related to the correlation coefficient between W and W_{proxy}, r, under the assumptions of the linear model as:

$$\alpha = \beta_1 = r \, \frac{\sigma_{W_{proxy}}}{\sigma_W}$$

For an allele to behave as effectively neutral under idealized population genetic conditions (i.e., constant population size at equilibrium), the relationship between the selection coefficient ($s = 1 - W$) and the effective population size (N_e; $\sim 10^4$ in humans [98]) is:

$$s \ll \frac{1}{2N_e}$$

Based on this relationship, we can estimate the lower bound on the scaling factor relating W_{proxy} to W that would be consistent with neutral evolution of the aneuploidy risk allele:

$$s = \frac{1}{2N_e}$$

$$(1 - W) = \frac{1}{2N_e}$$

$$1 - \left(\alpha W_{proxy} + (1 - \alpha)\right) = \frac{1}{2N_e}$$

$$\alpha\left(1 - W_{proxy}\right) = \frac{1}{2N_e}$$

$$\alpha = \frac{1}{2N_e s_{proxy}}$$

We note that this threshold is conditional on the underlying effect-size β for the aneuploidy risk allele, where stronger effects on aneuploidy require weaker correlation between number of euploid embryos and fitness to be compatible with neutral evolution. The threshold also depends on the limits of the reproductive window, particularly the upper bound. For example, for an upper bound of 30 years (averaged over human evolutionary history), we require $\alpha \approx 9.43 \times 10^{-3}$ to be consistent with neutral evolution of the risk allele (**Fig. 5B**).

Supplementary Figures

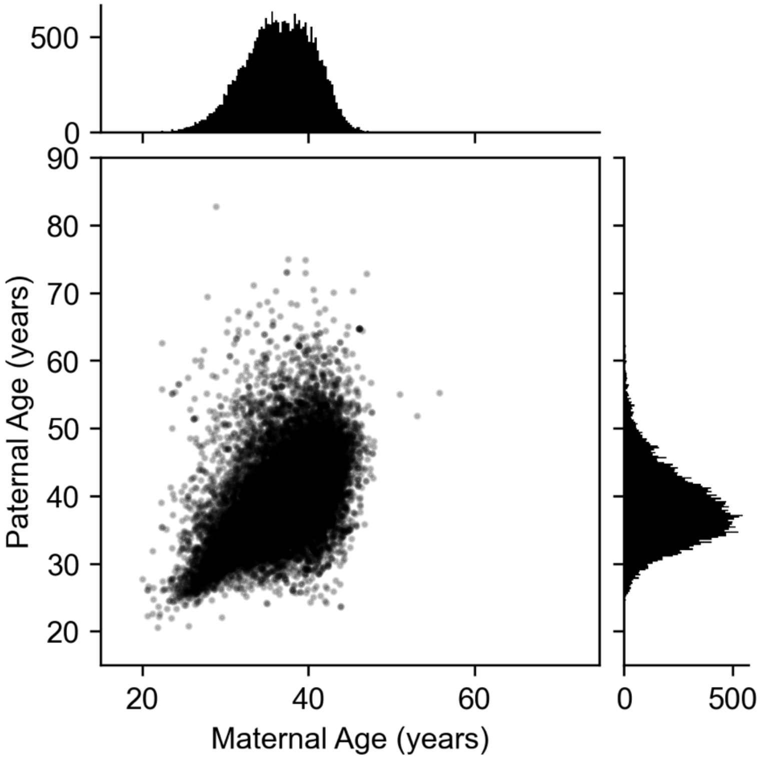

Figure S1. Age at time of collection across 22,850 sets of biological parents, excluding egg and sperm donors.

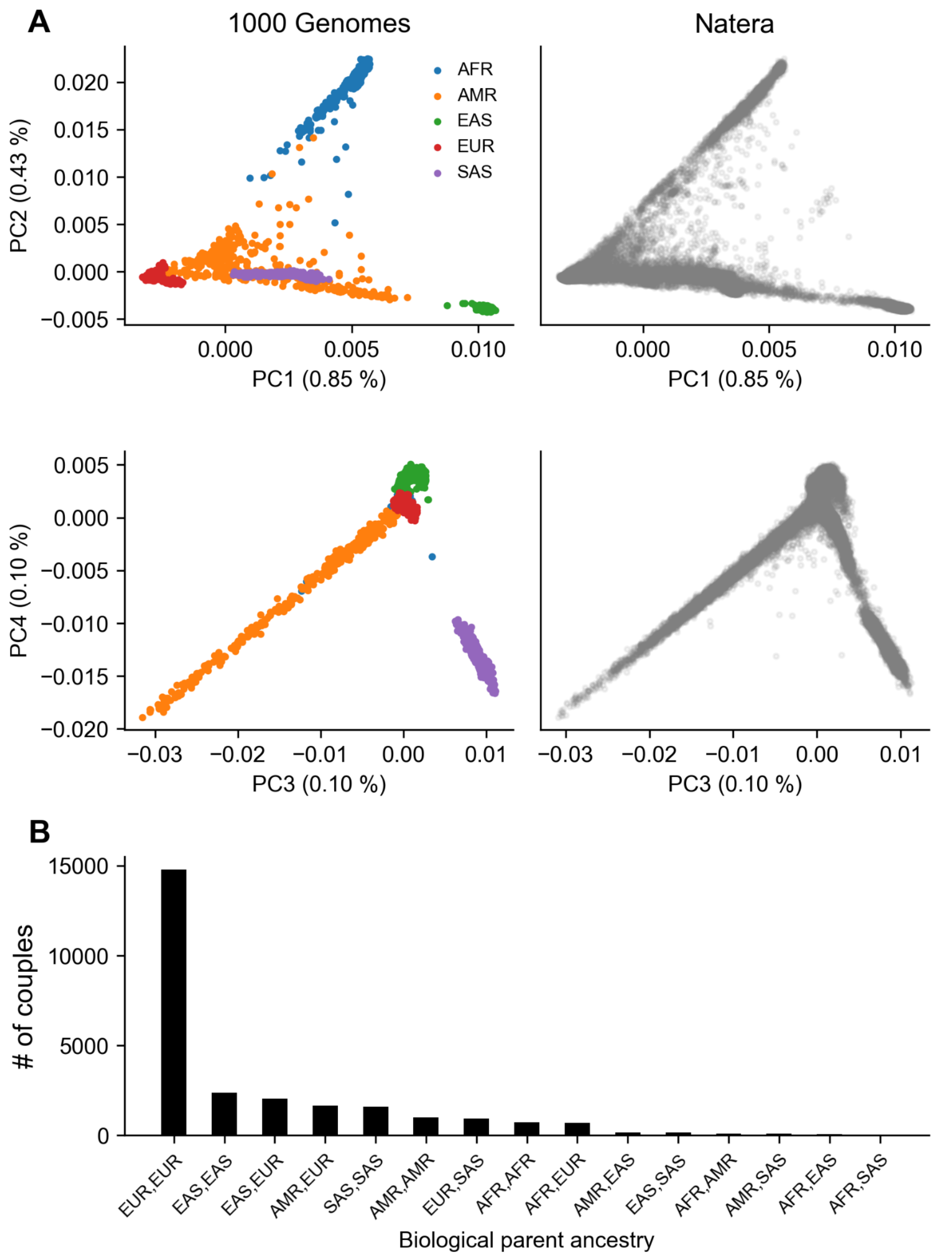

Figure S2. Population structure and ancestry inference of biological parents in the Natera datasets. (A) Joint PCA with 1000 Genomes individuals at shared polymorphic sites on the HumanCytoSNP array for only participants and partners, excluding embryos where genotypes cannot be reliably assigned. (B) Ancestry of biological parents, where labels reflect increased genetic similarity to reference individual population labels from the 1000 Genomes Project regional groupings using nearest-neighbors in PCA-space.

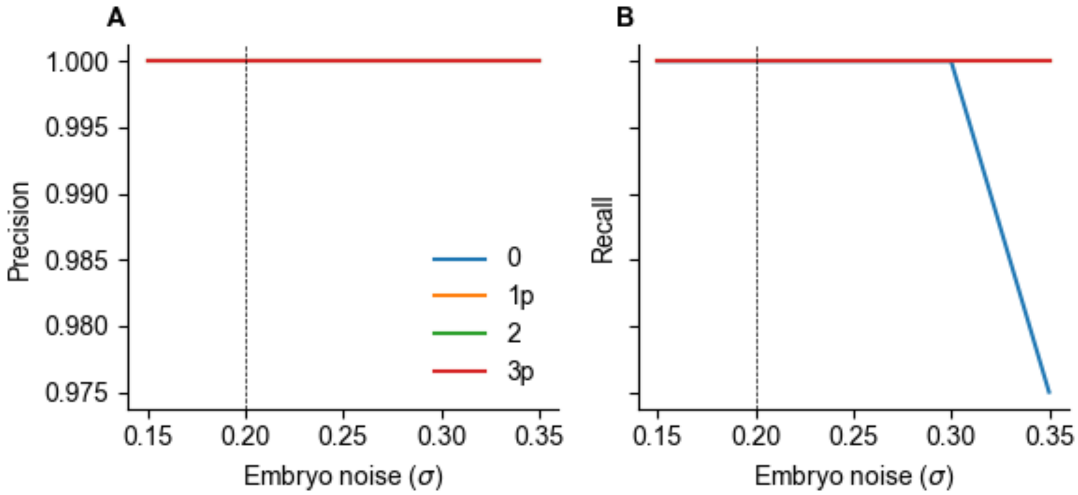

Figure S3. Performance of the karyoHMM method to detect simulated meiotic aneuploidies. Dashed vertical line represents the median maximum-likelihood noise parameter across all real embryos.

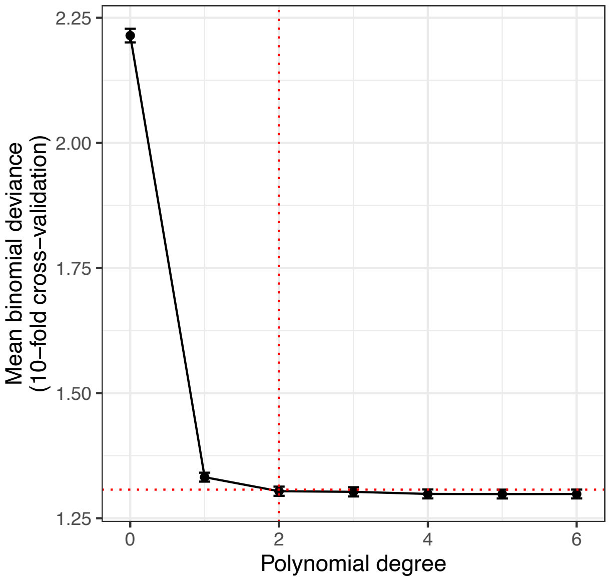

Figure S4. Mean binomial deviance across 10 cross-validation folds for nested quasibinomial models relating counts of embryos affected versus unaffected with maternal meiotic aneuploidy to increasingly complex polynomial predictor terms for maternal age. Degree of 0 denotes an intercept-only model. Error bars represent ±1 standard error of the mean deviance.

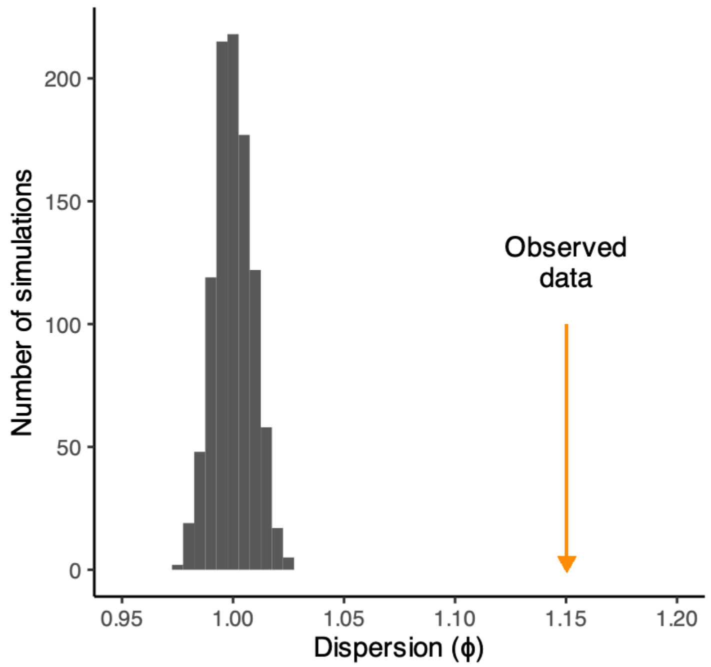

Figure S5: Dispersion parameters measured from data simulated under a binomial model (with no overdispersion), controlling for maternal age, compared to dispersion measured from the actual data. Dispersion is calculated as the sum of squared Pearson residuals, divided by the residual degrees of freedom.

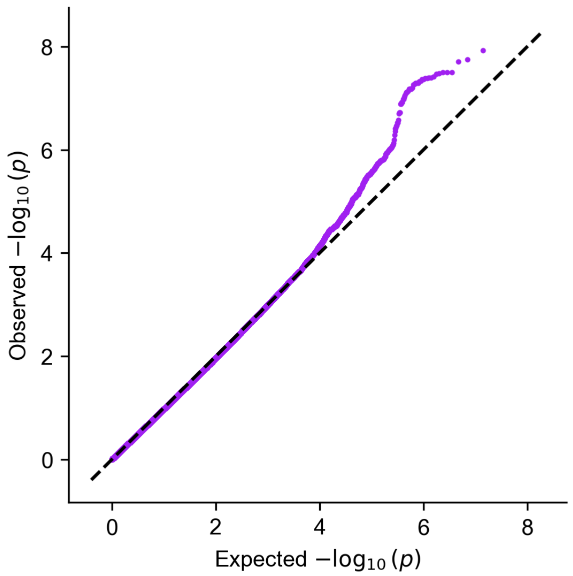

Figure S6. Quantile-quantile plot of GWAS for maternal meiotic aneuploidy.

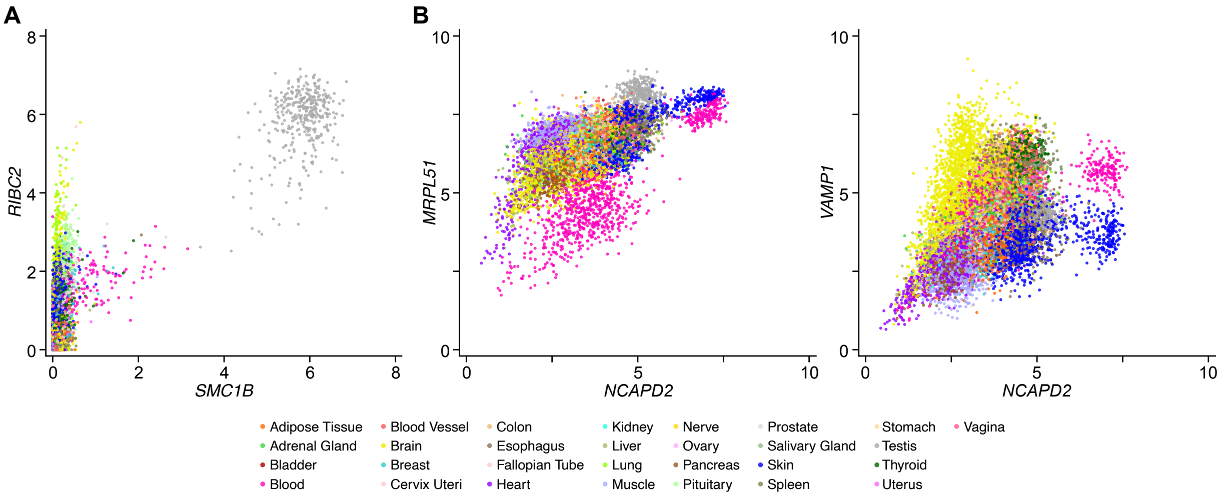

Figure S7. Genes associated with meiotic machinery are co-expressed. Scatter plots compare $\log_2$(TPM+1) gene expression levels from GTEx v8 RNA-seq [26] at two aneuploidy TWAS loci with multiple associated genes. Expression of (A) *RIBC2* with *SMC1B* at the first TWAS locus and (B) *VAMP1* and *MRPL1* with *NCAPD2* at the second TWAS locus are highly correlated.

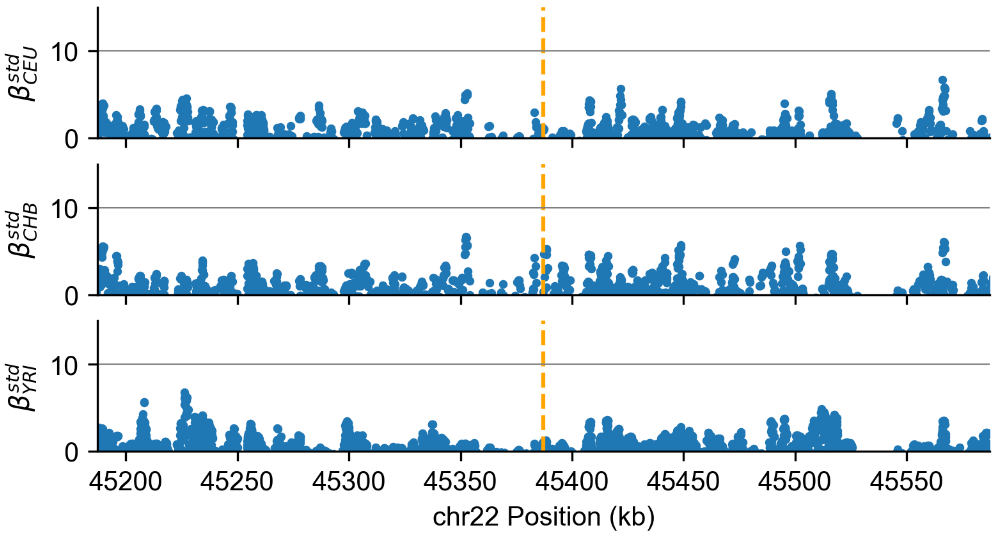

Figure S8. Estimates of the standardized beta statistic to identify balancing selection at the *SMC1B* locus. The threshold shows the empirical 99.9th percentile used to identify potential loci under balancing selection.

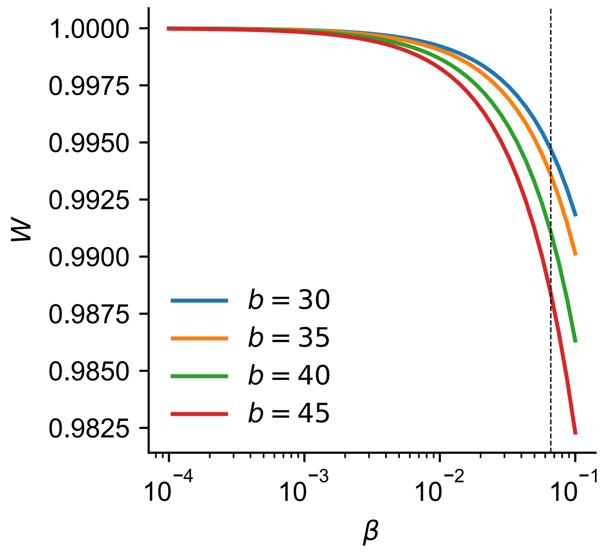

Figure S9. Estimated fitness of heterozygote carriers (w) of aneuploidy risk variants based on effect size (β) and stratified by upper bound of maternal reproductive window (b). The lower bound of the reproductive window is held constant at 18 years old in all cases.

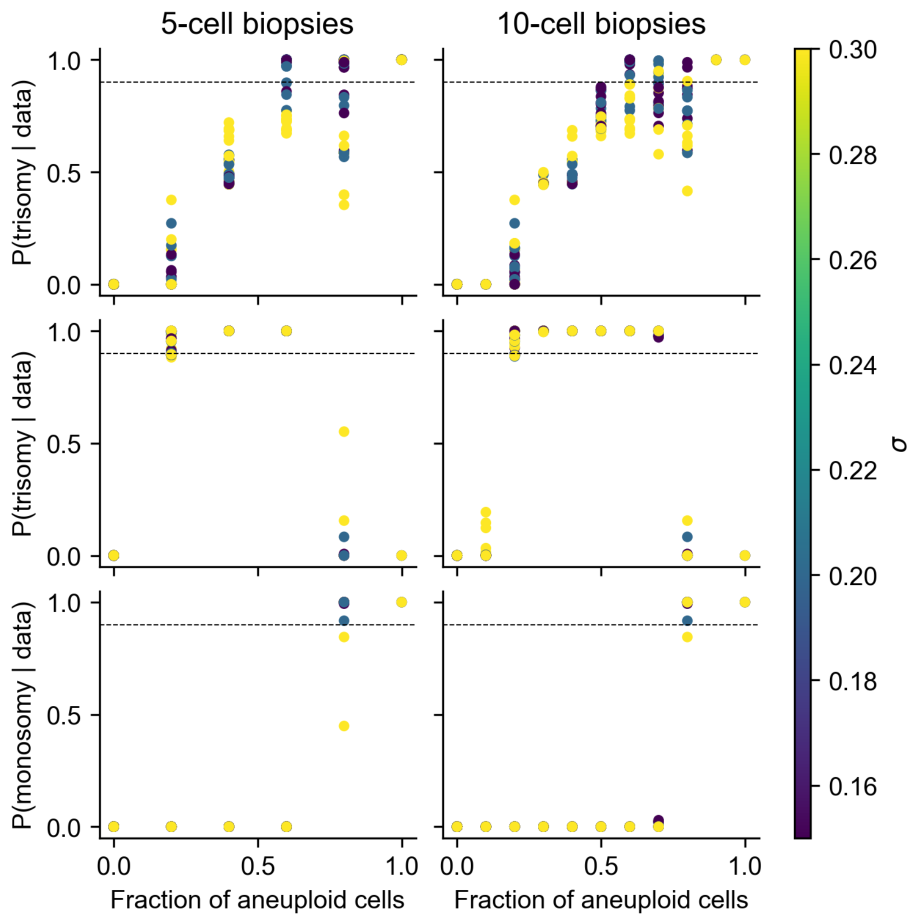

Figure S10. Posterior probability of trisomy across 5-cell (first column) and 10-cell biopsies at different levels of genotyping array noise. The first row shows an increasing cell fraction of mosaic trisomies. The second and third rows show the cell-fractions of simulated monosomies and their effects on the estimated posterior probability of trisomy and monosomy respectively.

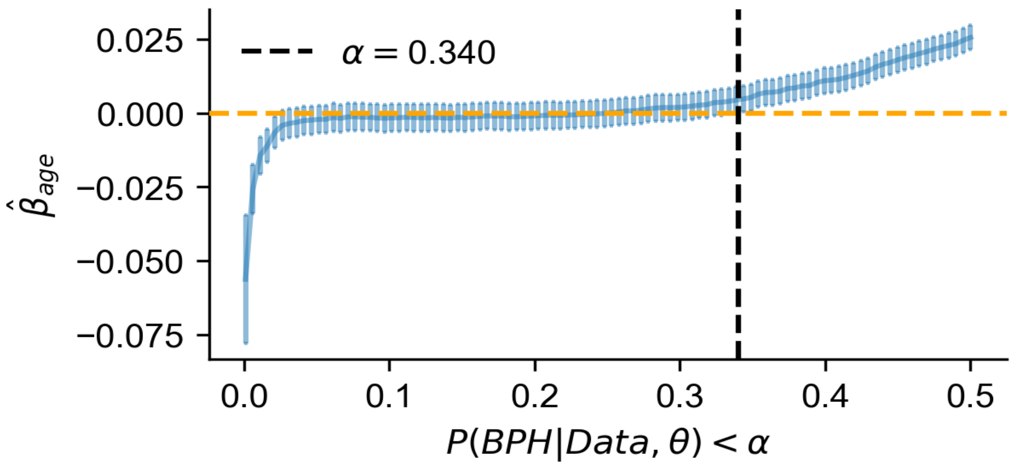

Figure S11. Maternal age effect size from binomial regression model treating mosaic trisomies as those with a posterior probability of BPH < α in the full dataset. Error bars reflect two standard errors.

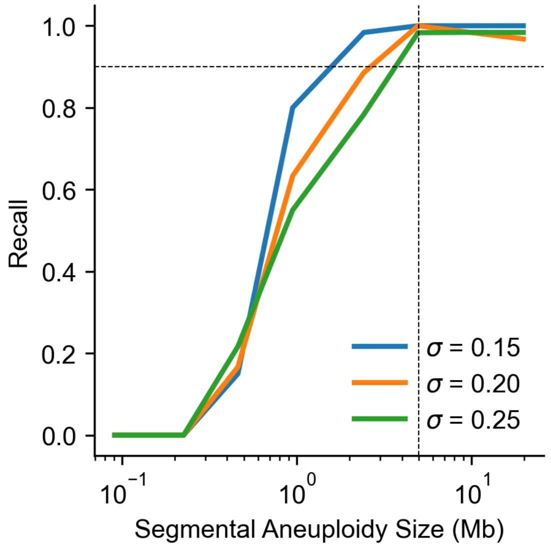

Figure S12. Mean recall across 50 simulated segmental aneuploidies at different lengths of segmental gains/ losses (equally distributed) and across increasing noise thresholds (σ). Vertical line indicates five Mbp threshold used for identifying segmental aneuploidies.

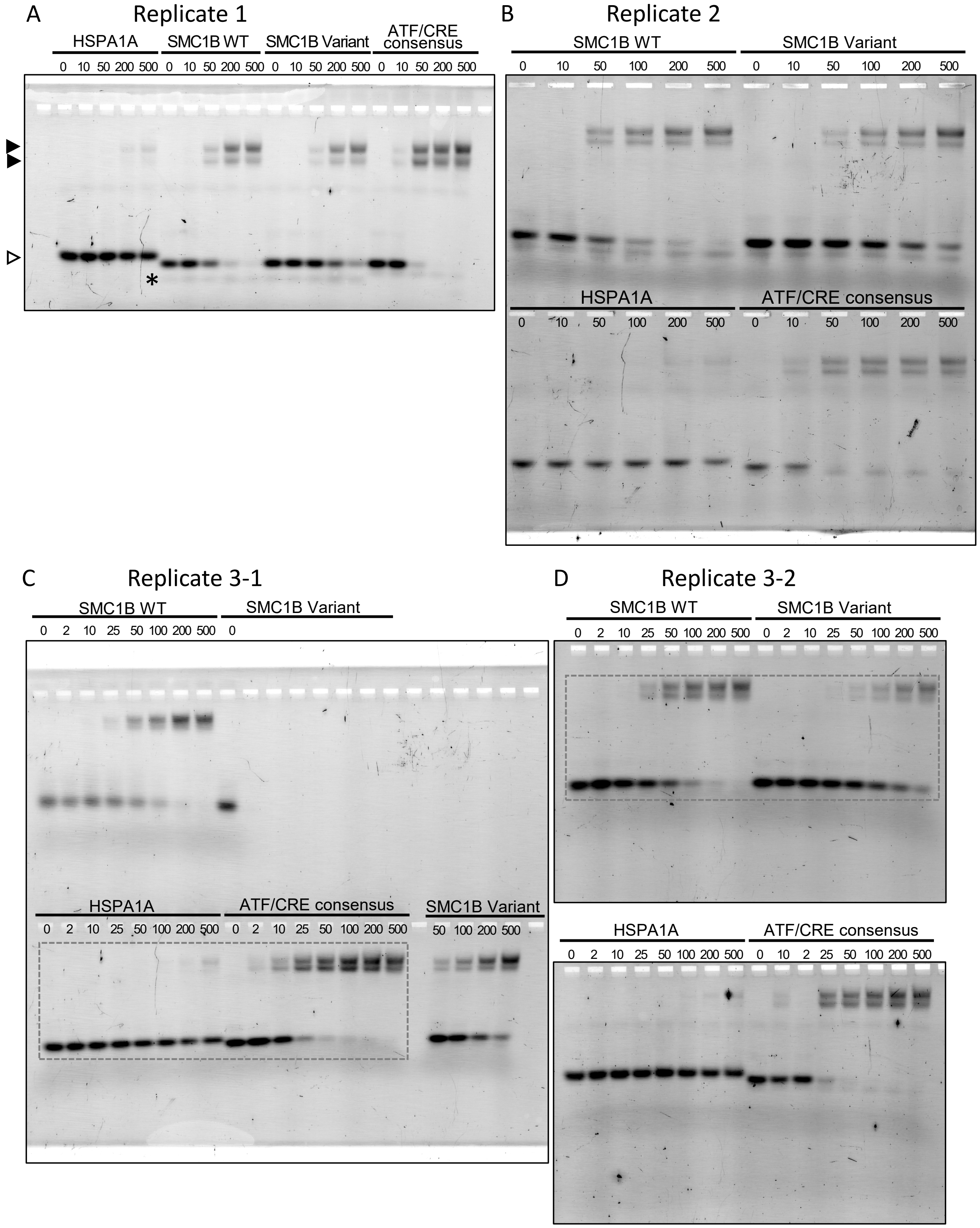
A
Replicate 1
HSPA1A
SMC1B WT
SMC1B Variant
ATF/CRE consensus
0 10 50 200 500 0 10 50 200 500 0 10 50 200 500 0 10 50 200 500
*
B
Replicate 2
SMC1B WT
SMC1B Variant
0 10 50 100 200 500 0 10 50 100 200 500
HSPA1A
ATF/CRE consensus
0 10 50 100 200 500 0 10 50 100 200 500
C
Replicate 3-1
SMC1B WT
SMC1B Variant
0 2 10 25 50 100 200 500 0
HSPA1A
ATF/CRE consensus
SMC1B Variant
0 2 10 25 50 100 200 500 0 2 10 25 50 100 200 500 50 100 200 500
D
Replicate 3-2
SMC1B WT
SMC1B Variant
0 2 10 25 50 100 200 500 0 2 10 25 50 100 200 500
HSPA1A
ATF/CRE consensus
0 2 10 25 50 100 200 500 0 10 2 25 50 100 200 500

Figure S13. Raw gel images for electrophoretic mobility shift assay (EMSA) demonstrating the impact of SNP rs2272804 on binding of transcription factor ATF1 in vitro across three independent replicates. EMSA gel scans for *SMC1B* reference and alternative sequences, ATF/CRE consensus sequence from the Epstein-Barr virus *LMP1* gene promoter as a positive control, and *HSPA1A* promoter sequence as a negative control across all three replicates.

References

1. Hassold, T. & Hunt, P. To err (meiotically) is human: the genesis of human aneuploidy. *Nat. Rev. Genet.* **2**, 280–291 (2001).

2. Gruhn, J. R. & Hoffmann, E. R. Errors of the egg: The establishment and progression of human aneuploidy research in the maternal germline. *Annu. Rev. Genet.* **56**, 369–390 (2022).

3. Baudat, F., Imai, Y. & de Massy, B. Meiotic recombination in mammals: localization and regulation. *Nat. Rev. Genet.* **14**, 794–806 (2013).

4. Hassold, T. J. & Hunt, P. A. Missed connections: recombination and human aneuploidy. *Prenat. Diagn.* **41**, 584–590 (2021).

5. Macklon, N. S., Geraedts, J. P. M. & Fauser, B. C. J. M. Conception to ongoing pregnancy: the “black box” of early pregnancy loss. *Hum. Reprod. Update* **8**, 333–343 (2002).

6. McCoy, R. C. *et al.* Meiotic and mitotic aneuploidies drive arrest of in vitro fertilized human preimplantation embryos. *Genome Med.* **15**, 77 (2023).

7. Handel, M. A. & Schimenti, J. C. Genetics of mammalian meiosis: regulation, dynamics and impact on fertility. *Nat. Rev. Genet.* **11**, 124–136 (2010).

8. Revenkova, E., Herrmann, K., Adelfalk, C. & Jessberger, R. Oocyte cohesin expression restricted to predictyate stages provides full fertility and prevents aneuploidy. *Curr. Biol.* **20**, 1529–1533 (2010).

9. Tachibana-Konwalski, K. *et al.* Rec8-containing cohesin maintains bivalents without turnover during the growing phase of mouse oocytes. *Genes Dev.* **24**, 2505–2516 (2010).

10. Hassold, T. *et al.* Failure to recombine is a common feature of human oogenesis. *Am. J. Hum. Genet.* **108**, 16–24 (2021).

11. Lamb, N. E. *et al.* Characterization of susceptible chiasma configurations that increase the risk for maternal nondisjunction of chromosome 21. *Hum. Mol. Genet.* **6**, 1391–1399 (1997).

12. Lister, L. M. *et al.* Age-related meiotic segregation errors in mammalian oocytes are preceded by depletion of cohesin and Sgo2. *Curr. Biol.* **20**, 1511–1521 (2010).

13. Gruhn, J. R. *et al.* Chromosome errors in human eggs shape natural fertility over reproductive life span. *Science* **365**, 1466–1469 (2019).

14. Halldorsson, B. V. *et al.* Characterizing mutagenic effects of recombination through a sequence-level genetic map. *Science* **363**, (2019).

15. Kong, A. *et al.* Recombination rate and reproductive success in humans. *Nat. Genet.* **36**, 1203–1206 (2004).

16. Ottolini, C. S. *et al.* Genome-wide maps of recombination and chromosome segregation in human oocytes and embryos show selection for maternal recombination rates. *Nat. Genet.* **47**, 727–735 (2015).

17. Bell, A. D. *et al.* Insights into variation in meiosis from 31,228 human sperm genomes. *Nature* **583**, 259–264 (2020).

18. Hinch, A. G. *et al.* Factors influencing meiotic recombination revealed by whole-genome sequencing of single sperm. *Science* **363**, eaau8861 (2019).

19. McCoy, R. C. *et al.* Evidence of Selection against Complex Mitotic-Origin Aneuploidy during Preimplantation Development. *PLoS Genet.* **11**, e1005601 (2015).

20. Warren, A. C. *et al.* Evidence for reduced recombination on the nondisjoined chromosomes 21 in Down syndrome. *Science* **237**, 652–654 (1987).

21. Lamb, N. E. *et al.* Susceptible chiasmate configurations of chromosome 21 predispose to non-disjunction in both maternal meiosis I and meiosis II. *Nat. Genet.* **14**, 400–405 (1996).

22. Ariad, D. *et al.* Aberrant landscapes of maternal meiotic crossovers contribute to aneuploidies in human embryos. *Genome Res.* **34**, 70–84 (2024).

23. Coop, G., Wen, X., Ober, C., Pritchard, J. K. & Przeworski, M. High-resolution mapping of crossovers reveals extensive variation in fine-scale recombination patterns among humans. *Science* **319**, 1395–1398 (2008).

24. Porubsky, D. *et al.* Human de novo mutation rates from a four-generation pedigree reference. *Nature* **643**, 427–436 (2025).

25. Kong, A. *et al.* Common and low-frequency variants associated with genome-wide recombination rate. *Nat Genet* **46**, 11–16 (2014).

26. GTEx Consortium. The GTEx Consortium atlas of genetic regulatory effects across human tissues. *Science* **369**, 1318–1330 (2020).

27. Gómez-H, L. *et al.* C14ORF39/SIX6OS1 is a constituent of the synaptonemal complex and is essential for mouse fertility. *Nat Commun* **7**, 13298 (2016).

28. Strong, E. R. & Schimenti, J. C. Evidence Implicating CCNB1IP1, a RING Domain-Containing Protein Required for Meiotic Crossing Over in Mice, as an E3 SUMO Ligase. *Genes (Basel)* **1**, 440–451 (2010).

29. Wang, S. *et al.* Per-Nucleus Crossover Covariation and Implications for Evolution. *Cell* **177**, 326-338.e16 (2019).

30. Hassold, T. J. A cytogenetic study of repeated spontaneous abortions. *Am. J. Hum. Genet.* **32**, 723–730 (1980).

31. Karczewski, K. J. *et al.* The mutational constraint spectrum quantified from variation in 141,456 humans. *Nature* **581**, 434–443 (2020).

32. Revenkova, E. *et al.* Cohesin SMC1 beta is required for meiotic chromosome dynamics, sister chromatid cohesion and DNA recombination. *Nat. Cell Biol.* **6**, 555–562 (2004).

33. Murdoch, B. *et al.* Altered cohesin gene dosage affects Mammalian meiotic chromosome structure and behavior. *PLoS Genet.* **9**, e1003241 (2013).

34. Taylor, D. J. *et al.* Sources of gene expression variation in a globally diverse human cohort. *Nature* **632**, 122–130 (2024).

35. Garcia-Alonso, L. *et al.* Single-cell roadmap of human gonadal development. *Nature* **607**, 540–547 (2022).

36. Roadmap Epigenomics Consortium *et al.* Integrative analysis of 111 reference human epigenomes. *Nature* **518**, 317–330 (2015).

37. Chitiashvili, T. *et al.* Female human primordial germ cells display X-chromosome dosage compensation despite the absence of X-inactivation. *Nat. Cell Biol.* **22**, 1436–1446 (2020).

38. ENCODE Project Consortium. An integrated encyclopedia of DNA elements in the human genome. *Nature* **489**, 57–74 (2012).

39. Xiong, M. *et al.* A common variant rs2272804 in the 5'UTR of RIBC2 inhibits downstream gene expression by creating an upstream open reading frame. *Eur. Rev. Med. Pharmacol. Sci.* **24**, 3839–3848 (2020).

40. Fan, S. *et al.* Homozygous mutations in C14orf39/SIX6OS1 cause non-obstructive azoospermia and premature ovarian insufficiency in humans. *Am J Hum Genet* **108**, 324–336 (2021).

41. Sánchez-Sáez, F. *et al.* Meiotic chromosome synapsis depends on multivalent SYCE1-SIX6OS1 interactions that are disrupted in cases of human infertility. *Sci. Adv.* **6**, (2020).

42. Schmiesing, J. A., Gregson, H. C., Zhou, S. & Yokomori, K. A human condensin complex containing hCAP-C-hCAP-E and CNAP1, a homolog of Xenopus XCAP-D2, colocalizes with phosphorylated histone H3 during the early stage of mitotic chromosome condensation. *Mol Cell Biol* **20**, 6996–7006 (2000).

43. Ruth, K. S. *et al.* Genetic insights into biological mechanisms governing human ovarian ageing. *Nature* **596**, 393–397 (2021).

44. Kentistou, K. A. *et al.* Understanding the genetic complexity of puberty timing across the allele frequency spectrum. *Nat. Genet.* **56**, 1397–1411 (2024).

45. Reynolds, A. *et al.* RNF212 is a dosage-sensitive regulator of crossing-over during mammalian meiosis. *Nat Genet* **45**, 269–278 (2013).

46. Day, F. R. *et al.* Large-scale genomic analyses link reproductive aging to hypothalamic signaling, breast cancer susceptibility and BRCA1-mediated DNA repair. *Nat. Genet.* **47**, 1294–1303 (2015).

47. Stankovic, S. *et al.* Genetic links between ovarian ageing, cancer risk and de novo mutation rates. *Nature* **633**, 608–614 (2024).

48. Venkatesh, S. S. *et al.* Genome-wide analyses identify 21 infertility loci and relationships with reproductive traits across the allele frequency spectrum. *Nat. Genet.* **57**, 1107–1118 (2025).

49. Albers, P. K. & McVean, G. Dating genomic variants and shared ancestry in population-scale sequencing data. *PLoS Biol.* **18**, e3000586 (2020).

50. Lawson, D. W. & Mace, R. Parental investment and the optimization of human family size. *Philos. Trans. R. Soc. Lond. B Biol. Sci.* **366**, 333–343 (2011).

51. Mathieson, I. *et al.* Genome-wide analysis identifies genetic effects on reproductive success and ongoing natural selection at the FADS locus. *Nat Hum Behav* **7**, 790–801 (2023).

52. Lande, R. & Arnold, S. J. The measurement of selection on correlated characters. *Evolution* **37**, 1210–1226 (1983).

53. Zhou, D., Zhou, Y., Xu, Y., Meng, R. & Gamazon, E. R. A phenome-wide scan reveals convergence of common and rare variant associations. *Genome Med.* **15**, 101 (2023).

54. Hou, D. *et al.* Variations of C14ORF39 and SYCE1 Identified in Idiopathic Premature Ovarian Insufficiency and Nonobstructive Azoospermia. *J Clin Endocrinol Metab* **107**, 724–734 (2022).

55. Mastrorosa, F. K. *et al.* Complete chromosome 21 centromere sequences from a Down syndrome family reveal size asymmetry and differences in kinetochore attachment. *bioRxiv* (2024) doi:10.1101/2024.02.25.581464.

56. Hinch, R., Donnelly, P. & Hinch, A. G. Meiotic DNA breaks drive multifaceted mutagenesis in the human germ line. *Science* **382**, eadh2531 (2023).

57. Forni, D., Mozzi, A., Sironi, M. & Cagliani, R. Positive selection drives the evolution of the structural Maintenance of Chromosomes (SMC) complexes. *Genes (Basel)* **15**, 1159 (2024).

58. Johnson, D. S. *et al.* Preclinical validation of a microarray method for full molecular karyotyping of blastomeres in a 24-h protocol. *Hum. Reprod.* **25**, 1066–1075 (2010).

59. Shah, T. S. *et al.* optiCall: a robust genotype-calling algorithm for rare, low-frequency and common variants. *Bioinformatics* **28**, 1598–1603 (2012).

60. Loh, P.-R. *et al.* Reference-based phasing using the Haplotype Reference Consortium panel. *Nat. Genet.* **48**, 1443–1448 (2016).

61. Koenig, Z. *et al.* A harmonized public resource of deeply sequenced diverse human genomes. *Genome Res.* **34**, 796–809 (2024).

62. Browning, B. L., Tian, X., Zhou, Y. & Browning, S. R. Fast two-stage phasing of large-scale sequence data. *Am. J. Hum. Genet.* **108**, 1880–1890 (2021).

63. Browning, B. L., Zhou, Y. & Browning, S. R. A One-Penny Imputed Genome from Next-Generation Reference Panels. *Am. J. Hum. Genet.* **103**, 338–348 (2018).

64. Huang, L. *et al.* Genotype-imputation accuracy across worldwide human populations. *Am. J. Hum. Genet.* **84**, 235–250 (2009).

65. Roach, J. C. *et al.* Analysis of genetic inheritance in a family quartet by whole-genome sequencing. *Science* **328**, 636–639 (2010).

66. Rabiner, L. R. A tutorial on hidden Markov models and selected applications in speech recognition. *Proc. IEEE* **77**, 257–286 (1989).

67. 1000 Genomes Project Consortium *et al.* A global reference for human genetic variation. *Nature* **526**, 68–74 (2015).

68. O’Connell, J. *et al.* A general approach for haplotype phasing across the full spectrum of relatedness. *PLoS Genet.* **10**, e1004234 (2014).

69. McCoy, R. C. Mosaicism in preimplantation human embryos: When chromosomal abnormalities are the norm. *Trends Genet.* **33**, 448–463 (2017).

70. Viotti, M. *et al.* Using outcome data from one thousand mosaic embryo transfers to formulate an embryo ranking system for clinical use. *Fertil. Steril.* **115**, 1212–1224 (2021).

71. Ariad, D. *et al.* Haplotype-aware inference of human chromosome abnormalities. *Proc. Natl. Acad. Sci. U. S. A.* **118**, (2021).

72. Ma, Y. *et al.* Mapping of meiotic recombination in human preimplantation blastocysts. *G3 (Bethesda)* **13**, (2023).

73. Konstantinidis, M. *et al.* Aneuploidy and recombination in the human preimplantation embryo. Copy number variation analysis and genome-wide polymorphism genotyping. *Reprod. Biomed. Online* **40**, 479–493 (2020).

74. Hastie, T., Tibshirani, R. & Friedman, J. *The Elements of Statistical Learning: Data Mining, Inference, and Prediction, Second Edition*. (Springer, New York, NY, 2009).

75. Ding, Q. *et al.* The genetic architecture of DNA replication timing in human pluripotent stem cells. *Nat. Commun.* **12**, 6746 (2021).

76. Mbatchou, J. *et al.* Computationally efficient whole-genome regression for quantitative and binary traits. *Nat. Genet.* **53**, 1097–1103 (2021).

77. R Core Team. *R: A Language and Environment for Statistical Computing. R Foundation for Statistical Computing, Vienna*. (2024).

78. Bates, D., Mächler, M., Bolker, B. & Walker, S. Fitting linear mixed-effects models Usinglme4. *J. Stat. Softw.* **67**, (2015).

79. Liu, Y. *et al.* ACAT: A Fast and Powerful p Value Combination Method for Rare-Variant Analysis in Sequencing Studies. *Am J Hum Genet* **104**, 410–421 (2019).

80. Manichaikul, A. *et al.* Robust relationship inference in genome-wide association studies. *Bioinformatics* **26**, 2867–2873 (2010).

81. Tober, D., Garibaldi, C., Blair, A. & Baltzell, K. Alignment between expectations and experiences of egg donors: what does it mean to be informed? *Reprod. Biomed. Soc. Online* **12**, 1–13 (2021).

82. Fonseca, A. C. S., Barreiro, M., Tomé, A. & Vale-Fernandes, E. Male Reproductive Health - study of a sperm donor population. *JBRA Assist. Reprod.* **26**, 247–254 (2022).

83. Machiela, M. J. & Chanock, S. J. LDlink: a web-based application for exploring population-specific haplotype structure and linking correlated alleles of possible functional variants. *Bioinformatics* **31**, 3555–3557 (2015).

84. Byrska-Bishop, M. *et al.* High-coverage whole-genome sequencing of the expanded 1000 Genomes Project cohort including 602 trios. *Cell* **185**, 3426-3440.e19 (2022).

85. Han, S., Riyahi, S., Huang, X. & Kuhlwilm, M. A curated dataset of great ape genome diversity. *bioRxiv* (2025) doi:10.1101/2025.02.18.638799.

86. Prüfer, K. *et al.* A high-coverage Neandertal genome from Vindija Cave in Croatia. *Science* **358**, 655–658 (2017).

87. Mafessoni, F. *et al.* A high-coverage Neandertal genome from Chagyrskaya Cave. *Proc. Natl. Acad. Sci. U. S. A.* **117**, 15132–15136 (2020).

88. Prüfer, K. *et al.* The complete genome sequence of a Neanderthal from the Altai Mountains. *Nature* **505**, 43–49 (2014).

89. Barbeira, A. N. *et al.* Exploiting the GTEx resources to decipher the mechanisms at GWAS loci. *Genome Biol* **22**, 49 (2021).

90. Barbeira, A. N. *et al.* Fine-mapping and QTL tissue-sharing information improves the reliability of causal gene identification. *Genet Epidemiol* **44**, 854–867 (2020).

91. Bulik-Sullivan, B. K. *et al.* LD Score regression distinguishes confounding from polygenicity in genome-wide association studies. *Nat. Genet.* **47**, 291–295 (2015).

92. Karczewski, K. J. *et al.* Pan-UK Biobank GWAS improves discovery, analysis of genetic architecture, and resolution into ancestry-enriched effects. *bioRxiv* (2024) doi:10.1101/2024.03.13.24303864.

93. Bailey, T. L. & Gribskov, M. Combining evidence using p-values: application to sequence homology searches. *Bioinformatics* **14**, 48–54 (1998).

94. Sjöblom, A., Yang, W., Palmqvist, L., Jansson, A. & Rymo, L. An ATF/CRE element mediates both EBNA2-dependent and EBNA2-independent activation of the Epstein-Barr virus LMP1 gene promoter. *J. Virol.* **72**, 1365–1376 (1998).

95. Heffler, M. A., Walters, R. D. & Kugel, J. F. Using electrophoretic mobility shift assays to measure equilibrium dissociation constants: GAL4-p53 binding DNA as a model system. *Biochem. Mol. Biol. Educ.* **40**, 383–387 (2012).

96. Deng, Y., Nielsen, R. & Song, Y. S. Robust and accurate Bayesian inference of genome-

wide genealogies for large samples. *bioRxiv* (2024) doi:10.1101/2024.03.16.585351.

97. Siewert, K. M. & Voight, B. F. BetaScan2: Standardized statistics to detect balancing selection utilizing substitution data. *Genome Biol. Evol.* **12**, 3873–3877 (2020).

98. Kimura, M. Evolutionary rate at the molecular level. *Nature* **217**, 624–626 (1968).
