# Supplementary material for: Common variation in meiosis genes shapes human recombination and aneuploidy
Source: Nature. 2026 Jan 21;651(8104):146–53. doi: 10.1038/s41586-025-09964-2 (PMC12960220; doi:10.1038/s41586-025-09964-2)
Supplement: Supplementary file 2 — Reporting Summary [file 41586_2025_9964_MOESM2_ESM.pdf]

nature portfolio

Corresponding author(s): McCoy, Rajiv C.

Last updated by author(s): 10/24/2025

Reporting Summary

Nature Portfolio wishes to improve the reproducibility of the work that we publish. This form provides structure for consistency and transparency in reporting. For further information on Nature Portfolio policies, see our Editorial Policies and the Editorial Policy Checklist.

Statistics

For all statistical analyses, confirm that the following items are present in the figure legend, table legend, main text, or Methods section.

n/a	Confirmed	
☐	☒	The exact sample size (*n*) for each experimental group/condition, given as a discrete number and unit of measurement
☐	☒	A statement on whether measurements were taken from distinct samples or whether the same sample was measured repeatedly
☐	☒	The statistical test(s) used AND whether they are one- or two-sided<br>*Only common tests should be described solely by name; describe more complex techniques in the Methods section.*
☐	☒	A description of all covariates tested
☐	☒	A description of any assumptions or corrections, such as tests of normality and adjustment for multiple comparisons
☐	☒	A full description of the statistical parameters including central tendency (e.g. means) or other basic estimates (e.g. regression coefficient) AND variation (e.g. standard deviation) or associated estimates of uncertainty (e.g. confidence intervals)
☐	☒	For null hypothesis testing, the test statistic (e.g. *F*, *t*, *r*) with confidence intervals, effect sizes, degrees of freedom and *P* value noted<br>*Give P values as exact values whenever suitable.*
☐	☒	For Bayesian analysis, information on the choice of priors and Markov chain Monte Carlo settings
☐	☒	For hierarchical and complex designs, identification of the appropriate level for tests and full reporting of outcomes
☐	☒	Estimates of effect sizes (e.g. Cohen's *d*, Pearson's *r*), indicating how they were calculated

Our web collection on statistics for biologists contains articles on many of the points above.

Software and code

Policy information about availability of computer code

Data collection	No software was used for data collection, as data were received as raw X and Y genotype intensities for retrospective analysis.
Data analysis	Genotyping and imputation code is available on GitHub (https://github.com/mccoy-lab/natera_genotyping/) and archived on Zenodo (https://doi.org/10.5281/zenodo.17429676). Pipelines for inferring crossover recombination across sibling embryos is available on GitHub (https://github.com/mccoy-lab/natera_recomb) and archived on Zenodo (https://doi.org/10.5281/zenodo.17429678). Code for inferring aneuploidies and performing downstream analyses is available on GitHub (https://github.com/mccoy-lab/karyohmm; https://github.com/mccoy-lab/natera_aneuploidy) and archived on Zenodo (https://doi.org/10.5281/zenodo.17429669; https://doi.org/10.5281/zenodo.17429672).<br><br>Existing software packages used in the study include:<br>ACAT - version 0.91, https://github.com/yaowuliu/ACAT<br>BEAGLE - version 5.4, https://faculty.washington.edu/browning/beagle/b5_4.html<br>data.table - version 1.15.4, https://cran.r-project.org/package=data.table<br>Eagle - version 2.4.1, https://alkesgroup.broadinstitute.org/Eagle/<br>emmeans - version 1.10.3, https://rvlenth.github.io/emmeans/<br>LDlink - https://ldlink.nih.gov/<br>LD Score Regression, version 1.0.0 - https://github.com/bulik/ldsc<br>lme4 - version 1.1.35.5, https://cran.r-project.org/web/packages/lme4/index.html<br>MAST - version 4.11.4, meme-suite.org/<br>optiCall - version 0.8.1, www.well.ox.ac.uk/~gav/docs/optiCall/<br>PLINK - version 1.9, https://www.cog-genomics.org/plink/<br>R - version 4, https://www.r-project.org/

REGENIE - version 4.1, https://rgcgithub.github.io/regenie/
SINGER - version 0.1.8, https://github.com/popgenmethods/SINGER
tidyverse - version 2.0.0, https://tidyverse.org/

For manuscripts utilizing custom algorithms or software that are central to the research but not yet described in published literature, software must be made available to editors and reviewers. We strongly encourage code deposition in a community repository (e.g. GitHub). See the Nature Portfolio guidelines for submitting code & software for further information.

Data

Policy information about availability of data

All manuscripts must include a data availability statement. This statement should provide the following information, where applicable:

- Accession codes, unique identifiers, or web links for publicly available datasets
- A description of any restrictions on data availability
- For clinical datasets or third party data, please ensure that the statement adheres to our policy

Association study summary statistics and aneuploidy and crossover calls are available on Zenodo: https://doi.org/10.5281/zenodo.15114528. Patient privacy and data use restrictions do not allow for external deposition of raw SNP microarray data. Researchers may request access to these data from Natera by contacting Zachary Demko (zdemko@natera.com). Initial responses to these requests can be expected within one month of receipt. Approved requests will require separate data use agreements in compliance with appropriate privacy laws.

Public data resources used in the study include:
GRCh37, GRCh38 (https://hgdownload.soe.ucsc.edu/)
GENCODE v37 (https://www.gencodegenes.org/human/release_37.html)
gnomAD (https://gnomad.broadinstitute.org/)
MAGE (https://github.com/mccoy-lab/MAGE/)
GTEx v8 (https://github.com/broadinstitute/gtex-v8)
ENCODE (https://www.encodeproject.org/)
deCODE genetic map (https://www.science.org/doi/10.1126/science.aau1043 - Supplementary File 1)
1000 Genomes Project + HGDP reference panel (https://github.com/atgu/hgdp_tgp)

Research involving human participants, their data, or biological material

Policy information about studies with human participants or human data. See also policy information about sex, gender (identity/presentation), and sexual orientation and race, ethnicity and racism.

Reporting on sex and gender	Mapping of meiotic crossovers and genome-wide association studies were performed by stratifying on the sex of the biological parents who provided eggs or sperm for in vitro fertilization, prior to preimplantation genetic testing. We use the term "biological parents" to refer to this set of individuals, the term "female" to refer to the biological sex of individuals who provided eggs, and the term "male" to refer to the biological sex of the individuals who provided sperm. While analyses were applied to both groups, a greater focus was placed on the female samples, as most chromosome abnormalities originate during female meiosis (egg formation), as confirmed in our study.
Reporting on race, ethnicity, or other socially relevant groupings	Geographically-defined population labels (European, African) are used in reference to allele frequencies of variants in external databases such as gnomAD and the 1000 Genomes Project, as defined in the original studies. To examine the ancestry distribution of the Natera sample, we performed principal component analysis (PCA) and interpreted results based on genetic similarity to 1000 Genomes samples with geographically-defined population labels. We report these results in the context of genetic similarity to reference samples, which is the accurate interpretation of PCA.
Population characteristics	The age distribution of biological parents is provided in the "Sample overview" section of the Methods. Detailed clinical histories of the biological parents are not available, but potential reasons for ordering the Natera Spectrum preimplantation genetic test, as listed on the sample requisition form, include: Balanced translocation or insertion in normal individual (Q95.0); Cystic Fibrosis gene carrier (Z14.1); Encounter for male factor infertility in female patient (Z31.81); Female infertility, unspecified (N97.9); Family history of carrier of genetic disease (Z84.81); Genetic carrier status, other than Cystic Fibrosis (Z14.8); Other screening for genetic and chromosomal anomalies (Z13.79); Recurrent pregnancy loss (N96); and Other.
Recruitment	This was a retrospective analysis of existing data, so there was no prospective recruitment. The data source is described in the "Data collection and sampling" section of the Methods. Briefly, this is a cohort of patients undergoing preimplantation genetic testing for a variety of referral reasons, including reasons related to fertility diagnoses (see above), so results must be interpreted in context of this ascertainment bias.
Ethics oversight	Research by Johns Hopkins was reviewed by the Johns Hopkins Homewood IRB (Not Human Subjects Research determination), while Natera received approval from Salus IRB (Category 4 Exempt Research).

Note that full information on the approval of the study protocol must also be provided in the manuscript.

Field-specific reporting

Please select the one below that is the best fit for your research. If you are not sure, read the appropriate sections before making your selection.

☒ Life sciences ☐ Behavioural & social sciences ☐ Ecological, evolutionary & environmental sciences

For a reference copy of the document with all sections, see nature.com/documents/nr-reporting-summary-flat.pdf

Life sciences study design

All studies must disclose on these points even when the disclosure is negative.

Sample size	All available samples (after quality control) were used in the study to maximize statistical power.
Data exclusions	Exclusion criteria for individual analyses are described in detail in the Methods. Small proportions of samples were excluded as technical outliers, as conclusions drawn from such data were deemed unreliable. For example, embryos with five or more nullisomic chromosomes were excluded, as these likely reflect DNA amplification failures. Chromosomes with ploidy calls supported by posterior probabilities below 0.9 were excluded to improve confidence in downstream results. Patients with fewer than three embryos were excluded, as chromosome-scale phasing could not be reliably determined in such cases. These exclusion criteria were not pre-specified prior to the study but were established based on theoretical considerations (e.g., phasing logic), simulation, and/or exploratory data analyses of technical variable distributions. All filtering was performed prior to biological hypothesis testing (e.g., GWAS analyses).
Replication	Samples for the genome-wide association study were randomly split into an independent discovery (85%) and test (15%) set for internal replication.
Randomization	All groups contrasted in our study were derived from the data itself, controlling for relevant covariates, as detailed in the Methods. For example, genome-wide association analyses compared genotypes among individuals with low versus high rates of embryonic aneuploidy (encoded as an overdispersed binomial quantitative trait), controlling for covariates such as maternal age, genotype principal components, etc.
Blinding	Blinding was not applicable because no experimental groups were assigned. Genotype-phenotype associations were analyzed retrospectively using existing data.

Reporting for specific materials, systems and methods

We require information from authors about some types of materials, experimental systems and methods used in many studies. Here, indicate whether each material, system or method listed is relevant to your study. If you are not sure if a list item applies to your research, read the appropriate section before selecting a response.

Materials & experimental systems

n/a	Involved in the study	
☒	☐	Antibodies
☒	☐	Eukaryotic cell lines
☒	☐	Palaeontology and archaeology
☒	☐	Animals and other organisms
☒	☐	Clinical data
☒	☐	Dual use research of concern
☒	☐	Plants

Methods

n/a	Involved in the study	
☒	☐	ChIP-seq
☒	☐	Flow cytometry
☒	☐	MRI-based neuroimaging

Plants

Seed stocks	*Report on the source of all seed stocks or other plant material used. If applicable, state the seed stock centre and catalogue number. If plant specimens were collected from the field, describe the collection location, date and sampling procedures.*
Novel plant genotypes	*Describe the methods by which all novel plant genotypes were produced. This includes those generated by transgenic approaches, gene editing, chemical/radiation-based mutagenesis and hybridization. For transgenic lines, describe the transformation method, the number of independent lines analyzed and the generation upon which experiments were performed. For gene-edited lines, describe the editor used, the endogenous sequence targeted for editing, the targeting guide RNA sequence (if applicable) and how the editor was applied.*
Authentication	*Describe any authentication procedures for each seed stock used or novel genotype generated. Describe any experiments used to assess the effect of a mutation and, where applicable, how potential secondary effects (e.g. second site T-DNA insertions, mosiacism, off-target gene editing) were examined.*
